# 3D bioprinting for bile duct tissue engineering: current status and prospects

**DOI:** 10.3389/fbioe.2025.1554226

**Published:** 2025-04-14

**Authors:** Bo Gao

**Affiliations:** Department of Hepatobiliary Surgery, Affiliated Hospital of Hebei University, Baoding, China

**Keywords:** 3D bioprinting, bile duct tissue engineering, bioinks, regenerative medicine, vascularization

## Abstract

Bile duct disorders, including cholangiocarcinoma, primary sclerosing cholangitis, and iatrogenic injuries, pose significant clinical challenges due to limited regenerative capacity and the complexity of the biliary tree. In recent years, 3D bioprinting has emerged as a promising approach for bile duct tissue engineering by providing patient-specific geometries and facilitating the spatial organization of cells, scaffolding materials, and bioactive factors. This review presents a comprehensive overview of 3D bioprinting techniques for bile duct tissue engineering, focusing on fundamental principles, biomaterial selection, current achievements, key challenges, and future perspectives. We systematically discuss the latest technological breakthroughs, highlight emerging innovations such as organoid-based strategies and microfluidic-assisted 3D printing, and evaluate the prospects for clinical translation. Finally, we outline the main challenges—such as biocompatibility of materials, vascularization, immunological barriers, standardization of protocols, and regulatory hurdles—and propose directions for future research, emphasizing multidisciplinary collaboration and translational studies.

## 1 Introduction

The biliary system plays a pivotal role in the hepatic excretion of bile acids and various metabolic byproducts, with the intrahepatic and extrahepatic bile ducts ensuring efficient transport of bile to the gastrointestinal tract (Xia et al., 2021; Jablonska and Lampe, 2009). Pathological changes in the biliary tree-ranging from strictures, congenital anomalies, cholangiocarcinoma, primary sclerosing cholangitis, to iatrogenic injuries-can trigger a cascade of complications including cholestasis, secondary biliary cirrhosis, and eventual liver failure (Ohtsuka et al., 2014; Mocchegiani et al., 2023). Despite the availability of surgical interventions such as biliary reconstruction or liver transplantation, there remain significant challenges, including donor shortage, postoperative anastomotic strictures, graft rejection, and high morbidity (Nakanuma et al., 2001).

Tissue engineering and regenerative medicine present new strategies that combine biomaterial scaffolds, cells, and bioactive cues to promote tissue repair. In bile duct tissue engineering, the ultimate goal is to recreate a functional ductal conduit capable of sustaining luminal patency under physiological bile flow, supporting epithelial cell function, and integrating seamlessly with the host environment (Hamada et al., 2021). The field of 3D bioprinting has garnered growing interest for precisely building complex tissue constructs. By integrating computer-aided design (CAD) and additive manufacturing, 3D bioprinting enables spatially controlled deposition of cells, biomaterials, and growth factors with high fidelity (Xiang et al., 2020). This capability is particularly relevant in addressing the unique structural intricacies of the bile duct, which combines a specialized epithelium (cholangiocytes), supportive fibromuscular layers, and an intricate vascular network (Farhat et al., 2021; Agarwal et al., 2021).

In this review, we examine the rationale for using 3D bioprinting in bile duct tissue engineering, elucidate the underlying principles of bioprinting technologies and biomaterial selection, and discuss various strategies for cellularization and *in vitro* maturation. We also highlight breakthroughs in in vivo models and provide a critique of the fundamental challenges that impede clinical application. Finally, we explore emerging areas such as microfluidic-assisted 3D bioprinting, 4D biomaterials, and organoid technology, offering insight into how these innovations may reshape the future of bile duct repair and regeneration.

## 2 Fundamentals of 3D bioprinting

### 2.1 Basic principles and workflows

3D bioprinting refers to the layer-by-layer deposition of cell-laden biomaterials (bioinks) to produce constructs that ideally recapitulate the 3D architecture and functionality of native tissues (Unagolla and Jayasuriya, 2020; Sorkio et al., 2018). This approach integrates biomaterials and bioactive molecules to fabricate tissue analogs, advancing regenerative medicine and tissue engineering (Hamid et al., 2021). The general workflow can be divided into four main stages:

#### 2.1.1 Digital design

Three-dimensional image data sets are required. CT and MRI image data generation initially consists of 2D image data sequences that have to be reconstructed into a 3D image data set using software (medical jargon: so-called 3D reconstructions). Depending on the type of device, there are different resolutions, so that artefacts can occur in the 3D reconstruction. For this and other reasons, a design software is needed.

When the patient-specific, individual 3D image data set is available, it must be translated into a G-code using special software. This is a machine language that is used to give instructions to a machine, in this case the 3D bioprinter. The more precise this G-code is, the better the printed construct will be, provided that the printer has the necessary mechatronic precision and performance.

In the case of bile duct engineering, the design might incorporate patient-specific dimensions or standardized geometries meant to mimic the extrahepatic or intrahepatic ductal shape. This is still the big challenge, because the image-based reconstruction of such complex anatomical structures and their transcription into G-code and printer precision and so on often have to be adjusted, since such anatomies in humans show strong dimensional variances due to natural variations and epigenetic variations, etc.

#### 2.1.2 Bioink preparation

Bioink formulations typically include cells (e.g., primary cholangiocytes, iPSC-derived cholangiocytes), hydrogels (e.g., collagen, gelatin methacryloyl/GelMA, alginate), and other additives like growth factors or nanoparticles (Lewis et al., 2019). These biomaterials have previously been explored either specifically for bile duct tissue engineering or more generally for bioprinting tubular structures facing similar biological and mechanical challenges. For instance, type I collagen, despite being widely utilized, exhibits limited printability due to its mechanical and rheological properties, which complicate the bioprinting process (Diamantides et al., 2017). GelMA, though frequently employed due to its tunable mechanical characteristics and crosslinking capabilities, also presents challenges, particularly regarding compatibility with primary, terminally differentiated human cells (J et al., 2023). Additionally, while alginate is commonly used, selecting an appropriate formulation of alginate is critical for ensuring desirable biological functionality, as not all alginate variants support the specific cellular and physiological outcomes required (Piras and Smith, 2020).

The rheological properties of the bioink-viscosity, elasticity, shear-thinning behavior-must be tailored to the chosen printing modality (Cuvellier et al., 2021).

#### 2.1.3 Bioprinting process

Using the selected 3D printer (extrusion-based, inkjet, laser-assisted, or stereolithography), the prepared bioink is deposited according to the layer-by-layer instructions. Print fidelity depends on numerous parameters such as print speed, nozzle diameter, crosslinking mechanisms, and environmental conditions (temperature, humidity) (Persaud et al., 2022).

#### 2.1.4 Post-processing and tissue maturation

Printed constructs are often crosslinked chemically, ionically, or via UV/visible light. They are then cultured in static or dynamic systems that facilitate nutrient delivery, waste removal, and mechanical cues. Over time, cells proliferate, secrete extracellular matrix (ECM), and potentially differentiate into specialized cell types, ideally forming functional tissue. The optimization of crosslinking strategies is critical for maintaining structural fidelity and promoting cellular behavior such as adhesion, proliferation, and differentiation (Zhang et al., 2023).

### 2.2 Classification of 3D bioprinting techniques

#### 2.2.1 Extrusion-based bioprinting

Extrusion-based methods employ pneumatic or mechanical pressure to continuously expel bioink from a nozzle. This approach is popular due to its ability to handle relatively high-viscosity bioinks, which can be advantageous in printing mechanically robust tubular constructs like bile ducts. However, its resolution and accuracy may be lower than with other modalities, potentially impacting fine structural features of the ductal epithelium (Rossi et al., 2024).

An advantage of extrusion-based printing for bile duct tissue engineering is the capacity to incorporate different cell types in consecutive layers or even within coaxial printing setups. But that would require two print reservoirs and print heads, which would then have to move to the corresponding position in succession. Coaxial extrusion, for instance, enables simultaneous deposition of two or more materials, one forming the luminal surface (e.g., cholangiocyte-laden hydrogel) and another forming an outer supportive layer (e.g., stroma-like hydrogel with fibroblasts) (Sun et al., 2023). This capability is especially useful in reconstructing the layered structure of the bile duct.

#### 2.2.2 Inkjet bioprinting

Inkjet bioprinting utilizes thermal or piezoelectric actuators to create droplets (2–50 pL) of cell-laden ink. The precise droplet ejection provides high throughput and minimal material waste. However, the low-viscosity requirement typically restricts the mechanical strength of the printed structures. Achieving a tubular geometry suitable for bile ducts may demand additional supportive scaffolds or temporary sacrificial inks (Sun et al., 2023). Furthermore, cholangiocytes can be sensitive to the thermal or mechanical forces experienced during droplet formation, so printing parameters must be carefully optimized (Derman et al., 2025).

Despite these limitations, inkjet printing excels in depositing multiple cell types or biomolecules in a spatially controlled manner, which can be leveraged to enhance epithelial differentiation or create chemical gradients mimicking *in vivo* environments (e.g., bile acid gradients) (Zhang et al., 2016).

#### 2.2.3 Laser-assisted bioprinting

Laser-assisted bioprinting (LAB) uses a pulsed laser beam to transfer droplets of bioink from a donor ribbon onto a receiving substrate. Without the need for a nozzle, LAB circumvents common issues like clogging and can print a broad range of cell densities and viscosities. The method provides excellent resolution (∼10–50 µm), beneficial for layering epithelial cells with high precision (Zhuang et al., 2023).

However, LAB setups can be expensive and require careful calibration to avoid heat-induced cell damage. These systems are also less common than extrusion-based printers and often have lower throughput, making them more suitable for small-scale research on bile duct epithelium rather than large-scale or clinically oriented manufacturing (Hakobyan et al., 2020).

The suitability of hydrogels for LIFT (Laser-Induced Forward Transfer)-based bioprinting depends critically on factors such as viscosity, gelation behavior, mechanical strength, thermal stability, and responsiveness to the energy imparted during laser transfer. Although a wide variety of hydrogels exhibit general biocompatibility, many are either unsuitable or remain unexplored specifically in the context of the LIFT bioprinting process. Future studies should systematically evaluate the compatibility and performance of a broader range of hydrogels with the LIFT technique, thus enabling a more comprehensive understanding and optimization of bioink formulations for complex tubular structures, including engineered bile ducts.

#### 2.2.4 Stereolithography and digital light processing

SLA/DLP printers selectively photopolymerize layers of resin-based or hydrogel-based materials using ultraviolet (UV) or visible light. By projecting patterns, these systems construct highly detailed, complex geometries with excellent surface finish. The resolution can be as fine as a few micrometers, which may facilitate intricate bile duct morphologies, including branching networks (Li et al., 2023).

One of the main drawbacks of SLA/DLP is the limited selection of photocurable bioinks. Photoinitiators must be cytocompatible, and unreacted monomers can pose toxicity risks. Nonetheless, recent developments in biocompatible resins and advanced photoinitiator systems are expanding the applicability of these techniques to bile duct tissue engineering (Huang et al., 2024).

Dynamic masks in DLP systems also allow the creation of gradient or region-specific polymerization intensities, potentially leading to functionally graded constructs that match the mechanical or biochemical heterogeneity found along the bile duct (Agarwal et al., 2023). It is manifested as enabling the generation of spatially controlled polymerization intensities, facilitating the fabrication of constructs with gradient or region-specific properties. This capability is particularly advantageous for engineering tissues like the bile duct, which exhibit inherent mechanical and biochemical heterogeneity along their length. By precisely tuning polymerization conditions, DLP technology can produce functionally graded constructs, mimicking the native gradients in stiffness, permeability, or bioactive molecule concentration. Such spatially tailored hydrogels not only enhance cellular function and differentiation but also support the formation of biologically relevant zonal architecture, improving the physiological fidelity of engineered bile duct tissues.

## 3 Material selection for bile duct bioprinting

### 3.1 Key considerations for bile duct tissue engineering

The native bile duct is a tubular organ lined by cholangiocytes, which have specialized junctional complexes (tight junctions, adherens junctions) for barrier function and active transport of bile acids (Shi et al., 2024). In extrahepatic bile ducts, supporting the epithelium is a lamina propria and a thin layer of smooth muscle cells, fibroblasts, and a modest vascular network. From a materials standpoint, the following factors are critical:

• Compatibility with Cholangiocytes
• Mechanical Behavior
• Chemical Stability
• Crosslinking and Printability
• Biodegradability and Biocompatibility

These requirements often necessitate composite bioinks that combine the cell-friendly nature of natural polymers (collagen, gelatin, alginate) with the mechanical robustness of synthetic polymers (PCL, PLGA, PEG) or other reinforcement strategies (Xiang et al., 2024).

### 3.2 Natural polymers

#### 3.2.1 Collagen and gelatin

Collagen type I is a major structural protein in many tissues, providing excellent cell adhesion sites (e.g., RGD motifs) and a native ECM environment. Gelatin, as a denatured product of collagen, is more soluble and can be chemically modified (e.g., GelMA) for tunable mechanical and degradation properties (Yue et al., 2015). However, pure collagen or gelatin hydrogels may exhibit suboptimal stiffness for tubular constructs under bile flow. Consequently, they are often blended with stiffer components (e.g., PCL microfibers) or crosslinked more extensively (Shi et al., 2024).

For bile duct applications, collagen-based hydrogels can support the differentiation of bile duct precursor cells and tight junction formation, which are vital for barrier integrity (Agarwal et al., 2021).

#### 3.2.2 Alginate

Alginate’s mild crosslinking using divalent cations (e.g., Ca^2+^) makes it appealing for cell encapsulation, preserving high cell viability (Tsou et al., 2016). However, alginate lacks native cell-adhesive domains, leading to poor cell attachment. Tailoring alginate by conjugating RGD peptides or blending with other ECM proteins can improve its biological functionality (Mohanty and Roy, 2024).

In bile duct engineering, alginate’s hydrophilic nature and tunable gelation can be advantageous for building tubular lumens. However, potential dissolution in alkaline environments and limited mechanical strength underscore the need for reinforcement.

#### 3.2.3 Chitosan

Chitosan exhibits antibacterial properties and can be chemically functionalized via its free amine groups (Shachar et al., 2011). However, its application in bile duct bioprinting remains in earlier stages, partly because chitosan’s solubility profile is pH-dependent, and it may precipitate in alkaline conditions. More research is needed to refine chitosan-based hydrogels for ductal applications, particularly by improving their mechanical stability and compatibility with bile duct environments.

### 3.3 Synthetic polymers

#### 3.3.1 Polycaprolactone

Polycaprolactone (PCL) stands out for its superior mechanical properties, slow degradation rate, and established FDA approval in certain medical devices. For bile duct constructs, PCL can serve as an external skeleton that confers structural stability, while the lumen is lined with a more cell-friendly hydrogel (Balagangadharan et al., 2019). Nonetheless, PCL’s hydrophobic nature and absence of bioactive cues necessitate surface modifications or co-printing with proteins and peptides to enhance cellular interactions.

#### 3.3.2 Poly (lactic-co-glycolic acid)

Poly (lactic-co-glycolic acid) (PLGA) degrades into lactic and glycolic acids, which are naturally metabolized in the body. The ratio of lactic to glycolic acid can be adjusted to fine-tune the degradation rate (Song et al., 2022). However, local acidification due to rapid breakdown can compromise the viability of cholangiocytes, making controlled-release strategies and buffering systems critical (Valderrama-Trevino et al., 2024). These strategies are particularly relevant in addressing the mechanical and biochemical requirements of bile duct tissue engineering.

#### 3.3.3 Polyethylene glycol

Polyethylene Glycol (PEG)-based hydrogels are prized for their tunable crosslinking, low immunogenicity, and well-defined chemical composition. By incorporating cell-adhesive motifs or ECM proteins, PEG can be tailored to support cholangiocyte growth (Maji and Lee, 2022). PEG alone lacks intrinsic bioactivity, necessitating blending with collagen or other natural polymers to enhance cellular interactions and mechanical properties.

### 3.4 Biomaterial combinations

The integration of biomaterial combinations aims to capture the best of both worlds: the biological affinity of natural polymers and the mechanical or chemical stability of synthetic polymers. For bile duct bioprinting, combinations like GelMA-PEG, PCL-PEG can be formulated to achieve an optimal balance of printability, mechanical strength, biocompatibility, and biodegradation (Liu et al., 2021; Cai et al., 2023).

## 4 Key technologies in bile duct tissue engineering

### 4.1 Cell sources and expansion

#### 4.1.1 Primary cholangiocytes and stem/progenitor cells

The functionality of the engineered bile duct largely depends on the viability and specialized phenotype of cholangiocytes. Primary cholangiocytes can be isolated from donor tissue but have limited expansion potential (Sun et al., 2021). Stem/progenitor cells, such as mesenchymal stem cells (MSCs) and induced pluripotent stem cells (iPSCs), offer scalable sources (Kim et al., 2023). Directed differentiation protocols typically incorporate growth factors (e.g., EGF, FGF) and small molecules (e.g., Notch modulators) to drive a cholangiocyte-like phenotype (Cotovio and Fernandes, 2020).

#### 4.1.2 Organoid technology

Organoid systems derived from cholangiocyte progenitors or iPSCs present a cutting-edge method to obtain functional biliary epithelial tissue *ex vivo*. Organoids can self-organize, forming hollow or cystic structures that closely resemble native ductal epithelium (Gan et al., 2023). Incorporating organoids into a printable bioink expands the potential to build tissues with pre-assembled epithelial architecture, offering enhanced scalability and precision for bile duct tissue engineering (Silva-Pedrosa et al., 2023).

### 4.2 Bioreactors and dynamic culture systems

Bioreactors designed for bile duct engineering typically feature fluid perfusion, adjustable shear stress, and hydrostatic pressure to mimic bile flow conditions. These parameters can promote alignment of cholangiocytes, enhance epithelial tight junction formation, and improve nutrient delivery (Juste-Lanas et al., 2023). Microfluidic-based perfusion systems can further refine fluidic parameters, enabling the investigation of bile acid transport and epithelial integrity (Rennert et al., 2015; Huang et al., 2020).

### 4.3 Vascularization strategies

Although the bile duct is not as highly vascularized as other tissues, a minimal capillary network is necessary to support metabolic needs. Integrating endothelial cells or endothelial progenitor cells into 3D-printed constructs can promote neovascularization, especially at the host-graft interface (Derman et al., 2025). Approaches such as coaxial extrusion of multiple cell types or incorporating growth factor–laden microspheres aim to accelerate angiogenesis (Li et al., 2024).

### 4.4 *In vitro* models and in vivo evaluation

Before proceeding to large-animal studies or clinical trials, *in vitro* models help refine material formulations and printing protocols. Advanced organ-on-a-chip platforms enable high-throughput screening of drug efficacy and mechanistic studies of cholangiopathy. *In vivo*, small-animal models (e.g., rats) are used to assess graft integration, patency, and function under physiological conditions (Haugabook et al., 2019). Larger animals such as pigs approximate human biliary anatomy more closely, allowing rigorous evaluation of feasibility and safety (Nair and Weiskirchen, 2023).

## 5 Current achievements in 3D bioprinted bile duct constructs

### 5.1 Proof-of-concept studies

Early work on 3D-printed bile duct conduits utilized simple tubular scaffolds composed of synthetic polymers like poly (ε-caprolactone) (PCL), seeded with primary cholangiocytes or biliary epithelial cell lines. These studies demonstrated the feasibility of forming a confluent epithelial layer *in vitro* and maintaining luminal integrity under flow (Justin et al., 2018). Advances in polymer chemistry and cell culture techniques have further enhanced the structural and functional outcomes of such constructs (Li et al., 2024).

### 5.2 Advanced multicellular constructs

Subsequent endeavors integrated supporting cell types such as fibroblasts and endothelial cells into the printed constructs to better mimic the native biliary niche. Coaxial extrusion printing has been employed to create dual-lumen designs, wherein the inner lumen is lined with cholangiocytes and the outer layer contains fibroblasts in a reinforcing hydrogel (Ashammakhi et al., 2019). Additionally, some studies have incorporated stem cells or iPSC-derived progenitors to enhance tissue remodeling and vascular integration (Shah Mohammadi et al., 2021; Hauser et al., 2021).

### 5.3 *In vivo* validation

Rodent models of bile duct injury have provided initial evidence that 3D-printed scaffolds can facilitate biliary regeneration and prevent stricture formation. Bioprinted grafts demonstrated partial recellularization by host cells, reduced fibrosis, and improved biliary drainage (Derman et al., 2025; Sun et al., 2021). Larger animal models, such as pigs, have also been used to assess long-term graft patency and immunogenicity, offering a closer approximation to human biliary anatomy and physiological conditions (Croce et al., 2019).

### 5.4 Organ-on-a-chip platforms

Bile duct-on-a-chip platforms allow fine control of fluid flow, bile acid composition, and drug exposure, providing insights into cholangiopathy and enabling personalized medicine approaches (Du et al., 2023). While not implantable, these platforms guide the optimization of bioink formulations, cell compositions, and perfusion conditions before *in vivo* studies (Tysoe et al., 2019). For instance, cell-printing has been used to develop a 3D liver-on-a-chip with multiple cell types for co-culture of liver cells, liver decellularized ECM bioink for a 3D microenvironment, and vascular/biliary fluidic channels for creating vascular and biliary systems (Lee et al., 2019).

## 6 Challenges in 3D bioprinting bile ducts

### 6.1 Material limitations

No single composition fully meets the mechanical, biological, and chemical requirements for long-term bile duct function (Funfak et al., 2019). Hydrogels that are conducive to cholangiocyte growth often lack the rigidity needed to withstand bile flow, while stiff synthetic scaffolds can degrade into acidic byproducts or hamper cell viability (Dvorakova et al., 2021). “Smart” materials that adapt to physiological environments are an emerging Frontier.

### 6.2 Achieving functional epithelialization

A viable bile duct construct must feature a continuous epithelial lining with strong cell-to-cell junctions and active transporter systems. In 3D bioprinting, cell viability can be compromised by shear stress, and post-printing differentiation may be incomplete. Approaches such as dynamic culture, co-cultivation with stromal cells, and controlled exposure to bile acids can enhance epithelial maturation (Zhuang et al., 2021). These strategies not only improve epithelial function but also mimic the physiological conditions necessary for long-term tissue integration.

### 6.3 Vascularization and integration

Adequate perfusion is essential to support the metabolic needs of cholangiocytes and surrounding fibrovascular tissue, particularly in thicker constructs. Incorporating endothelial cells, growth factor delivery, and sacrificial templates for vascular channels are strategies under investigation (Moreira et al., 2021). However, establishing robust anastomoses with host vasculature remains challenging.

### 6.4 Immunological and inflammatory responses

Any tissue-engineered graft can provoke an immune response, and the bile duct environment is further complicated by exposure to bile acids and potential microbial communities. Strategies to mitigate these responses include immunomodulatory biomaterials, use of autologous cell sources, and local immunosuppression (Petrosyan et al., 2022; Hussein et al., 2024). These approaches are critical for reducing graft rejection while maintaining tissue functionality.

### 6.5 Regulatory pathways and manufacturing scale-up

Regulatory approval for 3D-printed, cell-laden implants poses significant challenges due to the complexities of biological variability, manufacturing consistency, and device classification (Mladenovska et al., 2023). Standardizing protocols, ensuring GMP compliance, and conducting extensive preclinical safety studies are vital (Serrano et al., 2023). Collaboration between academia, industry, and regulatory agencies is needed to facilitate scalable, cost-effective bioprinting (Trubelja et al., 2022).

## 7 Future directions

### 7.1 4D bioprinting and stimuli-responsive materials

4D bioprinting introduces the dimension of time, enabling printed constructs to change shape or functionality in response to stimuli (Yarali et al., 2024). For bile duct engineering, shape-memory polymers or hydrogels that respond to pH or bile composition could facilitate better integration and reduce stricture formation.

### 7.2 Microfluidic-assisted bioprinting

Microfluidic-assisted 3D printing enables precise control over flow rates, gradients, and droplet formation, offering the possibility of spatially heterogeneous tissues with multiple cell types (Zhao et al., 2018). For bile duct fabrication, microfluidic nozzles can pattern cholangiocytes, smooth muscle cells, and fibroblasts in concentric layers.

### 7.3 Integration of omics and high-content analysis

Advanced omics (e.g., scRNA-seq) can elucidate the molecular underpinnings of differentiation to cholangiocytes and scaffold-induced immune responses (Mirhaidari et al., 2023). Such data-driven approaches can guide bioink design, dynamic culture conditions, and personalized graft development.

### 7.4 Organoid-based strategies

The rise of organoid technology presents powerful opportunities for bile duct engineering, given organoids’ capacity to recapitulate native epithelial phenotypes (Cabral et al., 2024). Embedding organoids into printable bioink matrices may drastically reduce the time required for post-printing maturation and improve epithelial function (Bernal et al., 2022).

### 7.5 Pathway to clinical translation

While preclinical studies show promise, factors such as long-term safety, surgical workflow, and cost-effectiveness must be addressed before clinical deployment. Early engagement with regulatory bodies and multidisciplinary collaboration are key to establishing guidelines for 3D-bioprinted bile duct constructs.

## 8 Conclusion

The application of 3D bioprinting to bile duct tissue engineering is still in a nascent stage but shows remarkable promise. Technological innovations in printing methods (extrusion, inkjet, laser-assisted, SLA/DLP), biomaterial design (composite hydrogels, stimuli-responsive polymers), and cell biology (organoid systems, iPSC-derived cholangiocytes) have collectively advanced our capacity to fabricate duct-like structures that mimic key functional traits of the native biliary epithelium.

While *in vitro* models and small animal studies indicate that engineered bile duct constructs can support partial regeneration and maintain luminal patency, multiple barriers remain. Material limitations, challenges in fully recapitulating epithelial architecture, vascularization strategies, and regulatory complexities must be tackled before clinical translation can be realized. The emergence of 4D bioprinting, microfluidic-assisted fabrication, and multi-omics-guided design will likely spur new solutions, enabling a more nuanced replication of bile duct geometry and cellular organization. Ultimately, sustained interdisciplinary collaboration is essential to transform these experimental advances into clinically deployable therapies. The potential impact—a decreased reliance on donor organs, reduced complication rates, and improved long-term outcomes for patients with biliary disease—highlights the imperative of ongoing innovation and translational rigor in this promising field.
